# The role of community health and nutrition volunteers in improving the health and nutrition status of infant and young children in remote areas, Hajjah, Yemen

**DOI:** 10.1186/s12887-024-04958-x

**Published:** 2024-07-27

**Authors:** Abdulkareem Ali Hussein Nassar, Ahmed Al-Haddad

**Affiliations:** Department of Community Medicine, Faculty of Medicine and Health Sciences, Sana’a University, Sana’a, Yemen

**Keywords:** Community health and nutrition volunteers, Volunteer and non-volunteer villages, Infant and young children, Health and nutrition status, Hajjah, Yemen

## Abstract

**Background:**

In Yemen, morbidity and malnutrition are major public health problems. The Community Health and Nutrition Volunteers (CHNVs) program was launched to tackle these problems through providing services to mothers and their children residing in remote villages. Since establishment of the CHNVs program in Yemen, its outcome has never been evaluated. Therefore, the aim of this study was to assess the role of CHNVs in improving the immunization, morbidity and nutritional status of infant and young children (IYC).

**Methods:**

A comparative cross-sectional study design was conducted in Al-Maghrabah and Bani-Qais districts, Hajjah governorate. It was carried out between January and April 2023. A three-stage cluster sampling method was used. A total of 926 IYC with their mothers were interviewed using a pre-tested questionnaire. SPSS 26 was used for data analysis. The multinomial logistic regression and chi-square or fisher exact tests were used to compare the vaccination, morbidity and nutritional status of IYC between the volunteer and non-volunteer villages. Odds Ratio (OR) with 95% Confidence Interval (CI) were calculated. A *p* value < 0.05 was considered statistically significant.

**Results:**

The IYC in volunteer villages were more likely to be fully or partially vaccinated compared to those in non-volunteer villages [OR = 2.3, 95% CI: 1.5–3.7, *p* < 0.0001, and OR = 1.9, 95% CI: 1.3–2.8, *p* = 0.001, respectively]. The specific coverage rates for BCG, and the 1st and 2nd doses of OPV/Pentavalent/Pneumo/Rota vaccines were significantly higher in the volunteer compared to non-volunteer villages [(OR = 1.8, 95% CI: 1.3–2.5, *p* < 0.0001), (OR = 1.5, 95% CI: 1.2–2.1, *p* = 0.003), and (OR = 1.5, 95% CI: 1.2-2.0, *p* = 0.002), respectively]. Moreover, the prevalence of diarrhea and fever among IYC was significantly lower in the volunteer compared to non-volunteer villages [(OR = 0.7, 95% CI: 0.5–0.9, *p* = 0.004) and (OR = 0.7 95% CI: 0.5–0.9, *p* = 0.045), respectively].

**Conclusions:**

The study found that CHNVs play a significant role in improving vaccination status and the coverage rate for BCG, and 1st and 2nd doses of OPV/Pentavalent/Pneumo/Rota vaccines, and reducing the prevalence of diarrhea and fever among IYC in their villages compared to non-volunteer villages, in Hajjah governorate. Future follow-up study and expansion to other settings in different governorates is recommended.

## Introduction

Globally, 149 million under-5 children were stunted and 45 million were wasted in 2020. Malnutrition is responsible for 45% of under-5 children deaths. These mostly occur in low- and middle-income countries [1]. Poor nutrition in early childhood impairs cognitive development, learning, adult educational attainment and economic productivity [2, 3]. However, proper and adequate nutrition for children helps ensure their growth, reinforces their immune system, improves their cognitive development, and reduces their risk of getting infectious diseases [2, 4–6].

The first 23 months of life is a critical period, because the growth, neurologic and immunologic development of children have adversely affected by malnutrition [1, 2, 6]. New global goals on maternal, and infant and young children (IYC) nutrition goals have been agreed for 2030 to reduce stunting by 50%, wasting to less than 3%, and overweight to not more than 3% in under-5 children. Support countries is needed to achieve global targets [7, 8]. Therefore, the World Health Organization (WHO) recommends expanding nutrition interventions within and beyond health facilities to improve nutrition [7].

One of strategies to address the shortage of human resources is to shift some tasks in health services to trained community members to expand access to basic health services and promote healthy behaviors at the community-level [9]. Community health workers (CHWs) tasks range from health promotion to disease prevention [10], nutrition counselling, management of acute malnutrition, micronutrient supplementation, growth monitoring and screening [7]. CHW programs are gaining importance due to their effectiveness in improving health outcomes, especially in resource-limited countries [11].

Although many published studies have assessed the outcomes of CHW activities, there’s no specific framework/model for their evaluation. Evaluation models could depend on resource availability, time constraints, and design considerations. Evaluations may focus on short-term, medium-term health changes (outcomes) or long-term results (impacts). Some studies compare baseline data to post-intervention data, while others compare intervention and control areas. For example, some studies showed that the CHWs can increase immunizations coverage [3, 12–15] and immunizations timeliness among children [12, 15, 16]. Other previous studies showed that the CHWs program can improve the nutritional status of children, such as stunting [2, 3, 17, 18], wasting [3, 19], underweight [2, 3, 19] and the mean weight and length of the infants [13]. Moreover, the intervention by CHWs can reduce child morbidity [13], such as diarrhea among IYC [4, 20], the mean number of days of diarrheal illness [21], and improve health care-seeking [3, 22].

Malnutrition in the Eastern Mediterranean Region (EMR) is a serious health problem, particularly in conflict-affected countries. Nearly 19.3% of babies born low birth weight, with rates exceeding 30% in Pakistan and Sudan, and 45% in Yemen [7]. In 2018, 20.2 million under-5 children (24.7%) were stunted and 6.4 million (7.8%) were wasted in the EMR [7]. In 2020, stunting prevalence in the EMR was 26.2%, while wasting prevalence was 7.4%. While stunting was highest in Libya (43.5%), Yemen (37.2%), and Pakistan (36.7%), wasting was highest in Djibouti (21.5%), Yemen (16.4%), and Sudan (16.3%) [23].

In Yemen, malnutrition is a major public health problem, causing high mortality, and affecting infants’ healthy life [24]. The 2013 Yemen National Health and Demographic Survey (YNHDS) reported a high prevalence of stunting (47%), wasting (16%) and underweight (39%) among under-5 children [25]. Moreover, many previous surveys have shown high rate of stunting, wasting, underweight, diarrhea, fever, cough, and symptoms of Acute respiratory infection (ARI) among children in Hajjah [25–27, 29, 30], Taiz [28], Al-Hodeidah [31], Sa’adah [32], and Dhamar governorates [33].

Yemen’s healthcare system faces the challenge of dispersing 70% of the rural population in scattered clusters. However, the Ministry of Public Health and Population (MPHP) has implemented the Community Health and Nutrition Volunteers (CHNVs) program to enhance healthcare accessibility. The CHNVs program provides service through female volunteers selected from villages of the 2nd and 3rd levels of the health facilities’ catchment areas (CHNVs Program, unpublished data, 2020). The CHNVs program is an extension of the Community Based Nutrition program that was launched by the MPHP to cover 244 villages in 10 districts with 371 volunteers in 2004 [34]. The number of CHNVs was gradually increased from 918 CHNVs at 35 districts in 2010 to 25,352 in 2019 at 243 districts of 21 governorates (CHNVs Program, unpublished data, 2020). Despite this expansion in recent years, the activities of CHNVs have never been evaluated in Yemen. The need for such study has become crucial in determining if the CHNVs program is effective, and the provided services contribute to improving the health and nutritional status among children, particularly the first 23 months of life. Moreover, the study will provide invaluable information to the MPHP officials and partners to gain insight on the CHNVs program activities. Therefore, the objective of this study was to assess the role of CHNVs in improving the immunization, morbidity and nutritional status of IYC in Hajjah governorate.

## Methods

### Study design, area and population

A comparative cross-sectional study was conducted among IYC and their mothers in Al-Maghrabah and Bani-Qais districts, Hajjah governorate, Yemen, from January to April 2023.

The outcome (potential effectiveness) of the CHNVs was assessed by comparing the immunization coverage, dropout and timeliness among IYC, nutritional status of IYC and their mothers, and morbidity prevalence among IYC between volunteer and non-volunteer villages.

### Inclusion and exclusion criteria

IYC aged 0–23 months with their mothers who had been living in the selected villages in Al-Maghrabah and Bani-Qais districts for the past year were included in this study. Mothers with children older than 23 months, or those who did not provide verbal consent, were excluded from this study.

### Sample size and sampling technique

A sample size of 454 IYC with their mothers from each district was determined based on assumptions and results from a pilot study, with an alpha error of 5% and a power of 95%. The G-Power software (version 3.1.9.4) was used to calculate the sample size, which was then increased by 5% to account for non-respondents. The final sample size was 476 IYC from 476 households (238 from volunteer and 238 from non-volunteer villages) for each district.

A three-stage cluster sampling approach was used to select districts, villages and households. In the first stage, districts were randomly selected. In the second stage, villages were randomly selected from each chosen district. In the third stage, households were randomly selected from each chosen village. A Probability Proportional to Size sampling method was used to select households from each volunteer village. For each IYC selected from a volunteer village, an age- and sex-matched IYC was enrolled from a non-volunteer village.

### Data collection

A pre-tested structured questionnaire has been adopted from previous literature [20, 25, 35–38]. The questionnaire included questions to elicit the information about the characteristics of IYC (age, sex, number of siblings and rank), and their mothers (age, education level, occupation status, attended antenatal care (ANC) and number of antenatal care visits, family size). Data on vaccine type, number of doses and date of vaccination were collected to estimate the overage and dropout rates, vaccination status and timely vaccination among IYC [25, 36, 38].

Data related to weight, height/length, Mid Upper Arm Circumference (MUAC) and oedema were collected to assess the nutritional status of IYC and their mothers using standardized methods [20, 35–37]. Moreover, data on diarrhea, fever and Cough/ARI were collected to assess the morbidity prevalence among IYC [20, 25, 35, 37]. Face-to-face interviews with mothers/caretakers were used to collect data. Anthropometric measurements were used to collect data on weight, and height/length.

The original questionnaire was translated into Arabic and back into English by two different centers of translation, and discrepancies were corrected.

A pilot study was carried out among 16 mothers from volunteer and non-volunteer villages. Any misunderstandings in the questions were revised and corrected to be more understandable and culturally appropriate.

### Definition and measurement of variables

The immunization status was estimated for all vaccinated children. IYC who have received all of the recommended vaccines and doses according to age are considered fully vaccinated. IYC who have received at least one, but not all, of the recommended vaccines and doses are considered partially vaccinated. IYC who have not received any of the recommended vaccines and doses are considered un-vaccinated. The vaccination coverage rate for the doses of specific vaccines was estimated for the age-eligible children according to the national immunization schedule’s recommended ages. Moreover, the vaccination dropout rate was estimated as general and specific vaccination dropout rates. The general vaccination dropout rate [Pentavalent dose 3 Measles-rubella vaccine dose 1 (MR1)] was assessed by calculating the proportion of IYC aged 12–23 months who were vaccinated with MR1 among those who received Pentavalent3. Specific vaccination dropout rate (Pentavalent vaccine 1–3) was assessed by estimating the proportion of IYC aged 5–23 months vaccinated with dose 3 of Pentavalent vaccine among those who were vaccinated with dose 1 of Pentavalent vaccine among IYC aged 5–23 months (Expanded National Program for Immunization, unpublished data, 2020).

The timeliness of vaccination was measured for children who had vaccination cards. On-time vaccination for specific vaccines is defined as vaccine dose administered within 4 days prior and within 4 weeks after the recommended age specified in the national immunization schedule. In contrast, untimely vaccination is defined as vaccine dose administered earlier (before 4 days) and/or delayed (after 4 weeks) than the recommended age specified in the national immunization schedule (if at least one vaccine dose was given early, late or missed at all considered as untimely vaccination) [38–40].

The Body mass index (BMI) (kg/m 2) was used to assess the nutritional status of women at reproductive age (15–49 years). Pregnant women during the survey were excluded from BMI calculations [25].

Furthermore, the nutritional status of IYC was estimated based on three anthropometry indices (z-scores). The weight-for-height z-score (WHZ) for IYC aged 6–23 months, height for age index z score (HAZ) for 6–23 month’s children and weight for age index z score (WAZ) for IYC aged 0–23 months. The WHZ (wasting), HAZ (stunting) and WAZ (underweight) were generated and compared with WHO 2006 Growth Standards. Additionally, the MUAC was used to estimate the nutritional status of IYC aged 6–23 months and classified based on the WHO cut-off-points [27, 30, 41].

Relating the morbidity indicators, diarrhea was estimated by asking mothers if their IYC had diarrhea (three or more loose or watery stools in a 24-hour period or any blood in the stools) in the preceding two weeks [35]. Fever was defined as (an abnormally high body temperature) and cough (as rapid expulsion of air from the lungs) were determined by the mother’s report [2]. The prevalence of ARI was estimated by asking mothers if their IYC had been ill during the last 2 weeks with a cough accompanied by short, rapid breathing, and/or by difficulty in breathing that was chest-related [25].

### Quality assurance and control during data collection and measurements

A supervisor and 4 female data collectors were trained on CHNVs program, nutrition, interviews, and data collection for two-days.

A non-stretchable standard MUAC tape to the nearest 0.1 cm was used with no clothing on the left arm. MUAC measurements were measured twice to ensure accuracy at midway between the elbow and shoulder of the left arm. The average of two measurements was calculated.

Moreover, an electric weight scale with 0.01 kg accuracy was used to measure the weight of IYC and their mothers. A standard infant length board with 0.1 cm accuracy was used to measure the length of IYC. A stadiometer to the nearest 0.1 cm was used to measure the height of mothers. The measurements of weight and length/height were repeated twice for every participant to ensure standardized anthropometric measurements.

### Statistical analysis

Data analysis was conducted using Statistical Package for the Social Sciences version 26 (SPSS 26). Based on the Shapiro-Wilk test (*p* < 0.05), the data of the quantitative variables were not normally distributed. However, the data were presented in medians and interquartile range (IQR) as quartile 1 and quartile 3. The frequency and percentage were used for categorical variables.

The WHO Anthro Survey Analyzer software v3.2.2 was used to calculate the Z-scores according to children’s sex, birth date, and survey date [42]. Based on the WHO flagging system, child/cases with extreme z-score values were flagged and investigated, and excluded from the final analysis [42]. Ten child/cases with extreme WHZ values ( > + 5 and <-5 z-score), 14 child/cases with extreme HAZ values ( > + 6 and <-6 z-score) and 6 child/cases with extreme WAZ values ( > + 5 and <-6 z-score) were also excluded from the final analysis [42].

The multinomial logistic regression analysis was used for more than two categorical variables. The chi-square or fisher exact tests were used for categorical variables. Fisher Exact test was used if the expected frequency in one or more cells of the contingency table was less than 5; otherwise, Chi-Square test was used. Odds Ratio (OR) with 95% Confidence Interval (CI) were calculated. A *p* value < 0.05 was considered statistically significant.

## Results

A total of 952 IYC with their mothers enrolled in this study from Al-Maghrabah and Bani-Qais districts in Hajjah governorate. Out of these, 926 completed the questionnaire, with response rates were 97%.

### Characteristics of participants

Table 1 shows the characteristics of participants by village type in Hajjah governorate. The characteristics of IYC and their mothers were comparable in volunteer and non-volunteer villages.

**Table 1:** Characteristics of the infant and young children, and their mothers by village type in Hajjah

Characteristics	Volunteer villages (*n* = 463)	Non-volunteer villages (*n* = 463)	Total (*n* = 926)
**Infant and young children**			
Age (months): median (IQR)	11.7 (7.3 and 17.2)	11.8 (7.1 and 17.0)	11.7 (7.2 and 17.1)
Sex: no (%)			
Male	242 (52.3)	242 (52.3)	484 (52.3)
Female	221 (47.7)	221 (47.7)	442 (47.7)
Number of siblings: median (IQR)	3.0 (1.0 and 5.0)	3.0 (1.0 and 5.0)	3.0 (1.0 and 5.0)
IYC rank: median (IQR)	4.0 (2.0 and 6.0)	4.0 (2.0 and 6.0)	4.0 (2.0 and 6.0)
**Mothers**			
Age (years): median (IQR)	25.0 (22.0 and 30.0)	25.0 (21.0 and 30.0)	25.0 (21.0 and 30.0)
Educational level: no (%)			
Not educated	344 (74.4)	395 (85.3)	739 (79.8)
Educated	119 (25.6)	68 (14.7)	187 (20.2)
Occupational status: no (%)			
Working	4 (0.9)	6 (1.3)	10 (1.1)
Not working	459 (99.1)	457 (98.7)	916 (98.9)
Attended antenatal care:			
Yes	250 (54.0)	270 (58.3)	520 (56.2)
No	213 (46.0)	193 (41.7)	406 (43.8)
Number of antenatal care visits: ^a^			
< 4 antenatal care visits	213 (85.2)	216 (80.0)	429 (82.5)
≥ 4 antenatal care visits	37 (14.8)	54 (20.0)	91 (17.5)
Family size: median (IQR)	6.0 (4.0 and 8.0)	7.0 (5.0 and 9.0)	6.0 (4.0 and 9.0)

IQR: interquartile range

^a^ Included the IYC’s mothers who attended antenatal care

### Vaccination characteristics of IYC

Table 2 shows the vaccination status, coverage and dropout rates, and timeliness among IYC by village type in Hajjah governorate. The percentage of fully and partially vaccinated IYC was significantly higher in volunteer villages (22.5% and 67.8%, respectively) compared to that in non-volunteer villages (17.5% and 64.8%, respectively). The OR for being fully and partially vaccinated were 2.3 (95% CI: 1.5–3.7, *p* < 0.0001), and 1.9 (95% CI: 1.3–2.8, *p* = 0.001), respectively. Moreover, the specific coverage rate of all vaccines among IYC was higher in the volunteer villages compared to non-volunteer villages, with the exception of MR vaccine. However, this difference was only statistically significant for BCG, and the 1st and 2nd doses of OPV/Pentavalent/Pneumo/Rota vaccines [(OR = 1.8, 95% CI: 1.3–2.5, *p* < 0.0001), (OR = 1.5, 95% CI: 1.2–2.1, *p* = 0.003), and (OR = 1.5, 95% CI: 1.2-2.0, *p* = 0.002), respectively].

**Table 2:** Vaccination characteristics of infant and young children by village type in Hajjah

Vaccination characteristics	Volunteer villages no (%)	Non-volunteer villages no (%)	Total no (%)	OR (95% CI)	*P* value
**Vaccination status**: ^**a**^	*n* = 463	*n* = 463	*n* = 926		
Fully vaccinated	104 (22.5)	81 (17.5)	185 (20.0)	2.3 (1.5–3.7)	< 0.0001^b^
Partially vaccinated	314 (67.8)	300 (64.8)	614 (66.3)	1.9 (1.3–2.8)	0.001^b^
Unvaccinated	45 (9.7)	82 (17.7)	127 (13.7)	Ref	
**Specific vaccine coverage rate**: ^**c**^					
BCG	391 (84.4)	348 (75.2)	739 (79.8)	1.8 (1.3–2.5)	< 0.0001^d^
OPV/Penta/Pneumo/Rota 1	343 (74.9)	302 (65.9)	645 (70.4)	1.5 (1.2–2.1)	0.003 ^d^
OPV/Penta/Pneumo/Rota 2	310 (68.7)	265 (58.9)	575 (63.8)	1.5 (1.2-2.0)	0.002 ^d^
OPV/Penta/Pneumo/Rota 3	251 (58.8)	223 (52.3)	474 (55.6)	1.3 (0.9–1.7)	0.058 ^d^
OPV4	132 (43.3)	119 (40.1)	251 (41.7)	1.1 (0.8–1.6)	0.424 ^d^
OPV5	18 (21.2)	17 (20.2)	35 (20.7)	1.1 (0.5–2.2)	0.880 ^d^
IPV1	249 (58.3)	234 (54.8)	483 (56.6)	1.2 (0.9–1.5)	0.300 ^d^
MR 1	197 (64.8)	203 (67.9)	400 (66.3)	0.9 (0.6–1.2)	0.422 ^d^
MR 2	32 (36.4)	34 (39.1)	66 (37.7)	0.9 (0.5–1.6)	0.711 ^d^
**Dropout rate**:					
No. of doses of pentavalent 1	309	263	572		
No. of doses of pentavalent 3	243	210	453		
**Specific Pentavalent 1–3 dropout**:	**66 (21.4)**	**53 (20.2)**	**119 (20.8)**	**0.9 (0.6–1.4)**	**0.723** ^**d**^
No. of doses of pentavalent 3	150	130	280		
No. of doses of MR1	123	121	244		
**General (Penta 3-MR1) dropout**:	**27 (18.0)**	**9 (6.9)**	**36 (12.9)**	**0.3 (0.2–0.8)**	**0.006** ^**d**^
**Vaccination timeliness for specific vaccines** ^**e**^					
BCG	22 (9.4)	19 (9.0)	41 (9.2)	1.1 (0.6-2.0)	0.861 ^d^
OPV/Penta/ Pneumo/Rota 1	204 (59.5)	205 (67.9)	409 (63.4)	0.7 (0.5–0.9)	0.027 ^d^
OPV/Penta/ Pneumo/Rota 2	146 (47.1)	136 (51.3)	282 (49.0)	0.8 (0.6–1.2)	0.313 ^d^
OPV/Penta/Pneumo/ Rota 3	94 (37.5)	84 (37.7)	178 (37.6)	0.9 (0.7–1.4)	0.961 ^d^
OPV 4	64 (48.5)	60 (50.4)	124 (49.4)	0.9 (0.6–1.5)	0.759 ^d^
OPV 5	5 (27.8)	8 (47.1)	13 (37.1)	0.4 (0.1–1.8)	0.238 ^d^
IPV 1	76 (36.2)	76 (39.0)	152 (37.5)	0.9 (0.6–1.3)	0.563 ^d^
MR 1	72 (45.0)	83 (52.9)	155 (48.9)	0.7 (0.5–1.1)	0.161 ^d^
MR 2	8 (32.0)	11 (50.0)	19 (40.4)	0.5 (0.1–1.5)	0.210 ^d^
Overall vaccination ontime	5 (1.4)	1 (0.3)	6 (0.9)	4.4 (0.5–38.1)	0.139 ^d^

BCG: Bacillus Calmette Guerin; OPV: oral polio vaccine; IPV: inactivated polio vaccine; MR: measles and rubella

^a^ Vaccination status was estimated according to age recommended in the national immunization schedule,

^b^ Multinomial regression analysis

^c^ Specific vaccine coverage rate was estimated based on the age-eligible IYC for specific vaccine dose according to age recommended in the national immunization schedule

^d^ Chi-square test or Fisher exact test

^e^ Included the age-eligible IYC for specific vaccine dose according to age recommended in the national immunization schedule and WHO recommended vaccination timelines, and those who have vaccination cards

The general Pentavalent 3-MR1 dropout rate was significantly higher in volunteer villages (18.0%) than that in non-volunteer villages (6.9%), (OR = 0.3, 95% CI: 0.2–0.8, *p* = 0.006).

Concerning the vaccination timeliness among IYC, there were no statistically significant differences in vaccination timeliness for all types and doses of vaccine between volunteer and non-volunteer villages, except for the timeliness of the 1st dose of OPV/Pentavalent/Pneumo/Rota vaccines (OR = 0.7, 95% CI: 0.5–0.9, *p* = 0.027), Table 2.

### Nutrition status of IYC and their mothers

Table 3 indicates the prevalence rate of malnutrition among IYC and their mothers by village type in Hajjah governorate. There were no statistically significant differences in the prevalence of malnutrition among IYC and their mothers between volunteer and non-volunteer villages (all *p* > 0.05).

**Table 3:** Nutrition status of infant and young children and their mothers by village type in Hajjah

Nutrition status	Volunteer villages no (%)	Non-volunteer villages no (%)	Total no (%)	OR (95% CI)	*P* value ^a^
**Infant and young children**					
Wasting (WHZ < -2 z-score and/or oedema):	*n* = 371	*n* = 365	*n* = 736		
Yes	50 (13.5)	42 (11.5)	92 (12.5)	1.2 (0.7–1.8)	0.419
No	321 (86.5)	323 (88.5)	644 (87.5)		
Stunting (HAZ < -2 z-score):	*n* = 370	*n* = 362	*n* = 732		
Yes	211 (57.0)	196 (54.1)	407 (55.6)	1.1 (0.8–1.5)	0.432
No	159 (43.0)	166 (45.9)	325 (44.4)		
Underweight (WAZ < -2 z-score):	*n* = 459	*n* = 461	*n* = 920		
Yes	177 (38.6)	167 (36.2)	344 (37.4)	1.1 (0.8–1.4)	0.464
No	282 (61.4)	294 (63.8)	576 (62.6)		
Acute malnutrition (MUAC < 125 mm and/or oedema):	*n* = 377	*n* = 369	*n* = 746		
Yes	136 (36.1)	128 (34.7)	264 (35.4)	1.1(0.8–1.4)	0.692
No	241 (63.9)	241 (65.3)	482 (64.6)		
**Mothers**					
Underweight (BMI < 18.5 kg/m^2^):	*n* = 448	*n* = 442	*n* = 890		
Yes	146 (32.6)	162 (36.7)	308 (34.6)	0.8 (0.6–1.1)	0.203
No	302 (67.4)	280 (63.3)	582 (65.4)		
Acute malnutrition (MUAC < 230 mm):	*n* = 463	*n* = 463	*n* = 926		
Yes	324 (70.0)	340 (73.4)	664 (71.7)	0.8 (0.6–1.1)	0.243
No	139 (30.0)	123 (26.6)	262 (28.3)		

WHZ: weight-for-height z-score; HAZ: height-for-Age z-score; WAZ: weight-for-age z-score; MUAC: mid upper arm circumference; BMI: body mass index

^a^ Chi-square test

### Morbidity prevalence among IYC

Table 4 shows the morbidity prevalence among IYC by village type in Hajjah governorate. The percentages of IYC who had diarrhea and fever in the two weeks preceding the interview were significantly lower in volunteer villages (53.3% and 73.2%, respectively), compared to non-volunteer villages (62.6% and 78.8%, respectively). The OR of having diarrhea and fever were 0.7 (95% CI: 0.5–0.9, *p* = 0.004) and 0.7 (95% CI: 0.5–0.9, *p* = 0.045), respectively.

**Table 4:** Morbidity prevalence among infant and young children by village type in Hajjah

Morbidity indicators	Volunteer villages no (%)	Non-volunteer villages no (%)	Total no (%)	OR (95% CI)	*P* value
**Morbidity**	*n* = 463	*n* = 463	*n* = 926		
Diarrhea:					
Yes	247 (53.3)	290 (62.6)	537 (58.0)	0.7 (0.5–0.9)	0.004 ^a^
No	216 (46.7)	173 (37.4)	389 (42.0)		
Fever:					
Yes	339 (73.2)	365 (78.8)	704 (76.0)	0.7 (0.5–0.9)	0.045 ^a^
No	124 (26.8)	98 (21.2)	222 (24.0)		
Cough:					
Yes	375 (81.0)	393 (84.9)	768 (82.9)	0.8 (0.5–1.1)	0.116 ^a^
No	88 (19.0)	70 (15.1)	158 (17.1)		
Symptoms of ARI:					
Yes	265 (70.7)	288 (73.3)	553 (72.0)	0.9 (0.8–1.2)	0.420 ^a^
No	110 (29.3)	105 (26.7)	215 (28.0)		
**Medical care seeking behavior**					
Seeking treatment for diarrhea: ^b^	*n* = 247	*n* = 290	*n* = 537		
Yes	154 (62.3)	199 (68.6)	353 (65.7)	0.8 (0.5–1.1)	0.127 ^a^
No	93 (37.7)	91 (31.4)	184 (34.3)		
Seeking treatment for fever: ^c^	*n* = 339	*n* = 365	*n* = 704		
Yes	338 (99.7)	364 (99.7)	702 (99.7)	0.9 (0.1–14.9)	1.000 ^d^
No	1 (0.3)	1 (0.3)	2 (0.3)		
Seeking treatment for cough/symptom of ARI: ^e^	*n* = 375	*n* = 393	*n* = 768		
Yes	373 (99.5)	384 (97.7)	757 (98.6)	4.4 (0.9–20.4)	0.064 ^a^
No	2 (0.5)	9 (2.3)	11 (1.4)		

ARI: acute respiratory infection

^a^ Chi-square test, ^b^ Included those who had diarrhea, ^c^ Included those who had fever, ^d^ Fisher exact test, ^e^ Included those who had cough/symptom of ARI

Although the percentages of IYC who had cough and symptoms of ARI in the two weeks preceding the interview were slightly lower in volunteer villages (81.0% and 70.7%, respectively), compared to non-volunteer villages (84.9% and 73.3%, respectively), these differences were not statistically significant (OR = 0.8, 95% CI: 0.5–1.1, *p* = 0.116), and (OR = 0.9, 95% CI: 0.8–1.2, *p* = 0.420), respectively.

Moreover, there were not statistically significant differences between those who sought advice/treatment for diarrhea, fever and cough/symptoms of ARI in volunteer and non-volunteer villages (all *p* > 0.05).

## Discussion

This study evaluated the outcome of the CHNVs by comparing health and nutrition status of IYC in volunteer and non-volunteer villages in Hajjah governorate, Yemen. It found that the CHNVs are effective in improving vaccination rates, and reducing the prevalence of diarrhea and fever among IYC.

Our findings indicated that the characteristics of IYC and their mothers are similar in volunteer and non-volunteer villages. This may be due to the study design matching IYC by age and sex, and comparable population structure in both types of villages.

Regarding vaccination status of IYC, findings of this study revealed that there is a positive association in vaccination status of IYC between volunteer and non-volunteer villages (*p* < 0.01). This means that the IYC in volunteer villages are 2.3 times more likely to be fully vaccinated and 1.9 times more likely to be partially vaccinated compared to those in non-volunteer villages. However, it seems that the role of CHNVs is not enough for improving vaccination coverage, and reducing the risk of IYC contracting infectious diseases. This is reflected by the fact that a significant number of IYC in both types of villages are still either unvaccinated or partially vaccinated. This is supported by two previous surveys, which showed that fully vaccinated children were low in Hajjah governorate [25, 30]. Our finding agrees with findings of three previous studies. The first one found that the percentage of children who completed their vaccination schedule in the first year of life was significantly higher in the intervention group than in the control group (*p* < 0.001) [14]. The second study found that there is a significant improvement in vaccination rate after a community-based child health counselling intervention (*p* < 0.001) [3]. The third study concluded that implementing community-based activity, such as CHWs, would have a positive impact on vaccination coverage, if their performance is continuously high [43].

This study found that there is a statistically significant association in the specific coverage rate for BCG, and the 1st and 2nd doses of OPV/Pentavalent/Pneumococcal/Rota vaccines among IYC between volunteer and non-volunteer villages. The IYC in volunteer villages are 1.8 times more likely to be vaccinated with BCG, and 1.5 times more likely to be vaccinated with the 1st and 2nd doses of OPV/Pentavalent/Pneumococcal/Rota vaccines compared to those in non-volunteer villages.

Nevertheless, the coverage rates for all specific vaccines in our study are less than 80%. These rates did not meet the Expanded Program on Immunization (EPI)’s recommended coverage rate of at least 90% at national level and 80% at all district levels [27]. This means that a significant number of IYC are at risk of contracting preventable diseases. This may be due to limited access to services in remote areas, lack of awareness, and rumors about vaccines. However, to improve vaccination coverage, there is still work to be done in these remote areas.

Our results in both types of villages are similar to results of previous surveys conducted in Hajjah and other governorates. These surveys found that the specific vaccine coverage rates are lower than the EPI’s recommended coverage rate for BCG [25], Diphtheria-tetanus-pertussis vaccine dose 3 [34], Pentavalent 3 [25, 30], OPV 3 [25–28, 32, 33], and measles vaccines [25, 28, 30, 32–34]. However, surveys in Hajjah and other governorates found that the specific coverage rate met the EPI’s recommended coverage rate for measles vaccine [26–28, 31] and OPV 3 [28, 31].

The current study revealed that there is no statistically significant difference in specific Pentavalent 1–3 dropout rate between volunteer and non-volunteer villages (*p* = 0.723). This suggests that CHNVs do not support EPI in tracking IYC to complete their vaccination schedule. This could be due to a lack of effective supervision from the health facilities. However, developing monthly lists of dropouts by the supervised health facilities and coordinating with CHNVs to track dropouts in their villages are needed to improve vaccination coverage rates.

Concerning vaccination timeliness, findings of this study found that there are no statistically significant differences in vaccination timeliness for vaccine types and doses between volunteer and non-volunteer villages. These findings disagree with the findings of three previous studies with a different design conducted in the United States (all *p* < 0.05) [12, 15, 16].

Moreover, our study found that there are no statistically significant differences in the prevalence of wasting, stunting, underweight, and acute malnutrition between IYC in the volunteer and non-volunteer villages (all *p* > 0.05). The results of this study do not support the expectation that CHNVs are effective in reducing nutritional problems among IYC in their villages. There are a number of possible explanations for this. First, factors like poverty, food scarcity, and limited healthcare access may affect intervention effectiveness in these remote communities. Second, there may be factors related to the CHNVs program itself, such as insufficient training, infrequent supervision, and irregular provision of micronutrient powders. Third, there may be factors related to CHNVs themselves, such as a lack of follow-up on referrals or monitoring of the growth of IYC. All of these factors could have affected the quality of CHNVs’ performance. Additionally, the study design matched IYC by age and sex could have affected the likelihood of detecting a statistically significant difference.

Our results agree with the findings of several previous studies that found no statistically significant differences in the prevalence of stunting [4, 14, 19, 20, 44–46], underweight [4, 14, 17, 20, 44–46], and wasting [2, 4, 14, 17, 18, 20, 45, 46] or in the mean weights in kg of infants [47] between children in the intervention and comparison groups. However, our results disagree with the findings of other previous studies that found statistically significant differences in the prevalence of stunting [2, 3, 17, 18], underweight [2, 3, 19], and wasting [3, 19] or in the mean weight and length of the infants [21, 48] between children in the intervention and comparison groups (all *p* < 0.05).

In both types of villages, our result found that the prevalence of stunting is somewhat similar to the range of 48–60% reported in previous surveys in Hajjah [25–27, 29, 30], and Al-Hodeidah governorates [31]. However, a higher prevalence of stunting is reported by two previous surveys, such as 64.7% in Sa’adah [32], and 66.2% in Dhamar governorates [33]. Another survey found a lower prevalence of stunting (44%) in Taiz governorate [28]. Such differences may be due to geographical and socioeconomic variations.

The overall prevalence of wasting in our study is higher (12.5%) than the target recommended by WHO which should be reduced to less than 3% [49]. Similarly, all the previous surveys in Hajjah [25–27, 29, 30], and other governorates [28, 31–33] have shown a higher prevalence of wasting than the WHO target. Strategies to tackle such high wasting figures are urgently needed.

In addition, the overall prevalence of underweight in our study is somewhat similar to the range of 35.1–48.0% reported in previous surveys in Hajjah [26, 27, 29, 30], Taiz [28], Sa’adah [32], and Dhamar governorates [33]. However, a higher prevalence of underweight is reported by two previous surveys, such as 55% in Hajjah [25], and 51.3% in Al-Hodeidah governorates [31].

This variance could be due to the fact that the prevalence estimation in the previous surveys was calculated for children aged 6–59 months and not for 6–23 months.

Contrary to expectations, this study’s findings do not indicate that CHNVs are effective in reducing nutritional problems among IYC’s mothers in their villages. Our study is consistent with two previous studies (all *p* > 0.05) [4, 44].

Furthermore, the study indicated that there is a statistically significant association in prevalence of diarrhea in the two weeks preceding the interview between volunteer and non-volunteer villages (*p* = 0.004). The prevalence of diarrhea among IYC is 30% less likely to be lower in the volunteer than non-volunteer villages. Our study agrees with two previous studies [4, 20], but disagrees with four previous studies [2, 14, 17, 46].

Overall, the prevalence of diarrhea among IYC in both types of village was 58% in this study. This result is higher than those reported by previous surveys in Hajjah, such as 49.0% [27], 38.9% [26], 38.7% [30] and 29.4% [25], and other governorates, such as 46.1% in Sa’adah [32], 41.7% in 2015 and 39.5%, in 2016 in Al-Hodeidah [31], 39.1% in Taiz [28], and 33.5% in Dhamar governorates [33].

Additionally, the current study revealed that there is a statistically significant association in prevalence of fever between volunteer and non-volunteer villages (*p* = 0.045). The prevalence of fever among IYC is 30% less likely to be lower in the volunteer than non-volunteer villages. Our study disagrees with three previous studies [2, 17, 46].

Overall, the prevalence of fever among IYC in both types of village was 76% in this study. This result is somewhat similar to a previous survey (67.6%) in Hajjah governorate [30]. However, other surveys indicated a lower prevalence of fever than ours, such as 57.7% [26], 59.4% [27] and 27.3% [25] in Hajjah, and 61.5% in Sa’adah [32], 47.7% in Al-Hodeidah [31], 53.8% in Taiz [28], and 46.5% in Dhamar governorates [33].

A significantly lower prevalence of diarrhea and fever in volunteer villages is possible due to the interventions that have been implemented by CHNVs such as counselling and education.

Furthermore, the present study indicated that the prevalence of cough, and symptoms of ARI among IYC is slightly more likely to be lower in the volunteer than non-volunteer villages, but these differences are not statistically significant. Our finding is similar to findings of previous studies, which found that no statistically significant difference in the prevalence of cough [2, 17, 46], the prevalence of difficulty breathing [17] between the study and control groups.

There are a number of possible explanations for the high prevalence of diarrhea, fever and cough, and symptoms of ARI among IYC in this study. Our study only included children under-2 years, who are more susceptible to diarrhea than older children. Furthermore, this study was conducted in remote areas with limited access to healthcare, low vaccination rates, poverty, poor sanitation, and infrequent handwashing. Moreover, the political conflicts that have been ongoing for over 10 years and the war that began in 2015 have worsened these factors, increasing the risk of infectious diseases.

Regarding medical care seeking behavior, the current study found that there are no statistically significant differences between those who sought advice/treatment for diarrhea, fever and cough/symptoms of ARI in the volunteer and non-volunteer villages.

Overall, the majority of respondents in both types of villages sought advice/treatment for diarrhea, fever and ARI. These findings are similar to two previous surveys in Hajjah [26] and Dhamar governorates [33]. However, a 2013 YNHDS indicated that the percentage of children for whom advice or treatment was sought for diarrhea, fever and symptoms of ARI was 20.5%, 20.3% and 22%, respectively [25].

The strengths of this study include a large, randomly selected and representative sample of IYC and their mothers from highland (Al-Maghrabah) and lowland (Bani-Qais) districts in Hajjah governorate. Moreover, the closest non-volunteer villages for comparison were selected from the same level of the health facilities’ catchment areas. This helps to control for potential confounding factors, such as socioeconomic status, access to healthcare or exposure to counselling or education. However, the study had some limitations. First, the study design, which matched IYC by age and sex, may have affected the likelihood of detecting a statistically significant difference. Secondly, the study was conducted in a single governorate, which may limit the generalizability of the findings. Third, the study used self-reported data on morbidity, which may be subject to recall bias. Fourth, the study did not collect data on the long-term impact of the CHNV program on health and nutritional status of IYC.

## Conclusions

The study found that CHNVs play a significant role in improving vaccination status, the coverage rate for BCG, and the 1st and 2nd doses of OPV/Pentavalent/Pneumo/Rota vaccines among IYC in their villages compared to non-volunteer villages, in Al-Maghrabah and Bani-Qais districts, Hajjah governorate. Moreover, they play a significant role in reducing the prevalence of diarrhea and fever among IYC in their villages compared to non-volunteer villages. However, the role of CHNVs is not enough to be effective in improving vaccination coverage among IYC, and tracking dropouts of IYC to complete their vaccination schedule and ensure timely vaccination. Additionally, CHNVs are not effective in reducing nutritional problems among IYC and mothers in their villages. A future study with a more robust study design and expansion to other settings in different governorates is recommended.

## Data Availability

No datasets were generated or analysed during the current study.

## Abbreviations

ANC: Antenatal care
ARI: Acute respiratory infection
BCG: Bacillus Calmette-Guerin Vaccine BMI: Body mass index
CHNVs: Community health and nutrition volunteers
CHWs: Community health workers
CI: Confidence interval
EMR: Eastern Mediterranean Region
EPI: Expanded Program on Immunization
HAZ: Height for age index z score
IPV: Inactivated polio vaccine
IQR: Interquartile range
IYC: Infant and young children
MPHP: Ministry of Public Health and Population
MR: Measles-rubella vaccine
MUAC: Mid upper arm circumference
OPV: Oral polio vaccine
OR: Odds ratio
ORS: Oral rehydration solution
WAZ: Weight for age index z score
WHO: World Health Organization
WHZ: Weight-for-height index Z score
YNHDS: Yemen National Health and Demographic Survey
